# Supplementary material for: Community-Based Approaches to Increase COVID-19 Vaccine Uptake and Demand: Lessons Learned from Four UNICEF-Supported Interventions
Source: Vaccines (Basel). 2023 Jun 30;11(7):1180. doi: 10.3390/vaccines11071180 (PMC10384848; doi:10.3390/vaccines11071180)
Supplement: Supplementary file 1 [file vaccines-11-01180-s001.zip › S1. Community survey _ Tool.pdf]

Gujarat Community survey

A. Basic Details

1. State

- ◯ Gujarat

2. District

- ◯ Mahesana
- ◯ Surat
- ◯ Banaskantha
- ◯ Sabarkantha
- ◯ Rajkot
- ◯ Ahmedabad

3. Block

4. Panchayat

5. Village

6. Tola/Hamlet

8. Community

- ◯ SC
- ◯ ST
- ◯ OBC
- ◯ General

9. Is the household from a DNT community?

- ◯ Yes
- ◯ No

10. Is the household from a Minority (Muslim) community? (Please ask the household members to reveal this information only if they are comfortable)

- ◯ Yes
- ◯ No
- ◯ No, from other minority community

10.1 In case from other minority community, please provide the name? (Please ask the household members to reveal this information only if they are comfortable)

11. Has the gram panchayat formulated any plan to deal with COVID?

- Yes plan already in place
- Yes developing plan
- No
- Don't know

12a. Please mention the top 3 main source of livelihood in the hamlet:

12b. Main livelihood source 1

- Daily wage Labour
- Agricultural Labour
- Brick Kiln
- Regular farming (paddy, vegetable)
- Plantation (spice, tea, coffee, cotton, sericulture, fruit/nut grove, sugarcane)
- Construction
- Textiles (garment factory, spinning mill, weaving, hand loom, dyeing unit)
- Regular salaried/wage employee in private sector
- Repairing and servicing (eg, of automobiles, electrical items)
- Traditional service occupation (cobbler, dhobi, barber)
- Regular salaried/wage employee in government
- Sex Work
- Leather industry
- Begging
- Others

If Others, please specify

12b. Main livelihood source 2

- ◯ Daily wage Labour
- ◯ Agricultural Labour
- ◯ Brick Kiln
- ◯ Regular farming (paddy, vegetable)
- ◯ Plantation (spice, tea, coffee, cotton, sericulture, fruit/nut grove, sugarcane)
- ◯ Construction
- ◯ Textiles (garment factory, spinning mill, weaving, hand loom, dyeing unit)
- ◯ Regular salaried/wage employee in private sector
- ◯ Repairing and servicing (eg, of automobiles, electrical items)
- ◯ Traditional service occupation (cobbler, dhobi, barber)
- ◯ Regular salaried/wage employee in government
- ◯ Sex Work
- ◯ Leather industry
- ◯ Begging
- ◯ Others

If Others, please specify

____

12b. Main livelihood source 3

- ◯ Daily wage Labour
- ◯ Agricultural Labour
- ◯ Brick Kiln
- ◯ Regular farming (paddy, vegetable)
- ◯ Plantation (spice, tea, coffee, cotton, sericulture, fruit/nut grove, sugarcane)
- ◯ Construction
- ◯ Textiles (garment factory, spinning mill, weaving, hand loom, dyeing unit)
- ◯ Regular salaried/wage employee in private sector
- ◯ Repairing and servicing (eg, of automobiles, electrical items)
- ◯ Traditional service occupation (cobbler, dhobi, barber)
- ◯ Regular salaried/wage employee in government
- ◯ Sex Work
- ◯ Leather industry
- ◯ Begging
- ◯ Others

If Others, please specify

____

13. In any of these main occupations, has there been any loss/drop in wages compared to last year (2020)

- ◯ Yes
- ◯ No

Migration

Is out-migration a common occurrence?

- ◯ Yes
- ◯ No

What is the primary occupation for which people migrate out?

- Daily wage Labour
- Agricultural Labour
- Brick Kiln
- Regular farming (paddy, vegetable)
- Plantation (spice, tea, coffee, cotton, sericulture, fruit/nut grove, sugarcane)
- Construction
- Textiles (garment factory, spinning mill, weaving, hand loom, dyeing unit)
- Regular salaried/wage employee in private sector
- Repairing and servicing (eg, of automobiles, electrical items)
- Traditional service occupation (cobbler, dhobi, barber)
- Regular salaried/wage employee in government
- Sex Work
- Leather industry
- Begging
- Others

If Others, please specify

____

Over the last one year, number of people involved in primary occupation have:

- Increased
- Decreased
- No change

Is there a migrant register being maintained at Panchayat level for listing out-migrants?

- Yes
- No
- Don't know

Ration and Access to Food (Nov-Jan)

Did people receive dry ration as per existing quota for November-January?

- All received
- Some received
- Negligable population or None received

For those that received ration, how was the quality of Ration in November-January?

- Good
- Average
- Poor

Were primary schools (till Class 5) running in November-January?

- Yes
- No

Were senior schools (Class 6-12) running in November-January?

- Yes
- No

For schools that were closed, did school going children (6 to 14 years) receive dry ration in lieu of mid-day meal during November-January?

- All received
- Some received
- Negligable population or None received

Did school going children (6 to 14 years) receive mid-day meal at school through November-January?

- Yes
- No

In your hamlet were pregnant women provided supplementary nutrition by the Anganwadi in November-January

- All received
- Some received
- Negligable population or None received
- No eligible Households

In your hamlet were lactating mothers provided supplementary nutrition by the Anganwadi in November-January

- All received
- Some received
- Negligable population or None received
- No eligible Households

In your hamlet were children between 6months-3years provided with Take Home Ration (THR) during November-January

- All received
- Some received
- Negligable population or None received
- No eligible Households

In your hamlet were children between 3-6years provided with a meal during November-January

- All received
- Some received
- Negligable population or None received
- No eligible Households

Did the 3 poorest HH in your hamlet receive dry ration through November-January?

- Do not have ration cards
- None of them received ration
- 1 of the HHs
- 2 of the HHs
- All 3 HHs received

Schemes and Entitlements (Nov-Jan)

For the people who have access to Ujjwala scheme, did they receive the cylinder in November-January

- None of them have access to the scheme
- All received
- Some received
- Negligable population or None received
- Not needed

Were eligible members able to receive Old Age Pension

- All received
- Some received
- Negligable population or None received
- No eligible Households

Were eligible members able to receive Widow Pension

- ◯ All received
- ◯ Some received
- ◯ Negligable population or None received
- ◯ No eligible Households

Were eligible members able to receive Disability Pension

- ◯ All received
- ◯ Some received
- ◯ Negligable population or None received
- ◯ No eligible Households

Have people in your hamlets got the Ayushman Bharat Health Insurance

- ◯ All received
- ◯ Some received
- ◯ Negligable population or None received
- ◯ No one has applied
- ◯ Do not know about scheme

Do people in the hamlet have bank accounts (including Jan Dhan and other micro bank accounts)

- ◯ All have account
- ◯ Some have account
- ◯ Negligable population or None have

Did the 3 poorest HH in the location get cylinders under Ujjwala scheme?

- ◯ None of them have access to the scheme
- ◯ None of them received the cylinder
- ◯ 1 of the HHs
- ◯ 2 of the HHs
- ◯ All 3 HHs received

Did the 3 poorest HH in the location get Old age/Widow/Disability Pension?

- ◯ Not eligible
- ◯ None of them
- ◯ 1 of the HHs
- ◯ 2 of the HHs
- ◯ All 3 HHs received

Do the 3 poorest HHs have Ayushman Bharat Health Insurance?

- Do not know about the scheme
- Have not applied
- None of them
- 1 of the HHs
- 2 of the HHs
- All 3 HHs received

Do the 3 poorest HHs have Banks Accounts (including Jan Dhan)?

- None of them
- 1 of the HHs
- 2 of the HHs
- All 3 HHs received

Were people able to access the e-nirman card?

- Do not know about this scheme
- All received
- Some received
- Negligable population or None received
- No eligible Households

MNREGA

Did people get MNREGA jobs in November-January

- All received
- Some received
- Negligable population or None received
- Not needed
- -

Were wages paid for work done in November-January

- Wages were paid in full to those who got jobs
- Only partial wages were paid to those who got jobs
- No wages were paid to those who got jobs
- Not Applicable

Infrastructure and Services

Total HHs covered under vaccination programme (100 or less)

____

Out these 100 houesholds, provide the number of HHs with access to safe drinking water

____

Out these 100 houesholds, provide the number of HHs with access to electricity

____

Out these 100 houesholds, provide the number of HHs with access to Toilets within the household

____

Out these 100 houesholds, provide the number of Pucca Households (cement walls and roof)

____

Out these 100 houesholds, provide the number of HHs where at least one person has a smart phone

____

Nearest internet/kiosk facility for submitting entitlement applications

- ○ Not existant
- ○ Accessible
- ○ Accessible and affordable

Are children able to access education through TV channels or online system?

- ○ All children
- ○ Some children
- ○ Neligable or no children

Are children from the 3 poorest HHs able to access online education?

- ○ None of them
- ○ 1 of the HHs
- ○ 2 of the HHs
- ○ All 3 HHs have access

Discrimination

During the last one year did you notice more incidences of domestic violence/physical abuse within families of your community?

- ◯ Yes
- ◯ No
- ◯ -
- ◯ Don't know

During the last one year did you notice more incidences of child abuse within families of your community?

- ◯ Yes
- ◯ No
- ◯ -
- ◯ Don't know

During the last one year has indebtedness increased in your community?

- ◯ Yes
- ◯ No
- ◯ -
- ◯ Don't know

Compared to other dominant caste/social group hamlets how would you assess the access to vaccination for your hamlet?

- ◯ Better
- ◯ Same
- ◯ Worse

Healthcare

Are people able to access services related to mental health support at government health facility?

- ◯ Yes
- ◯ No
- ◯ Don't know

Was immunisation of children carried out in November-January?

- ◯ All children
- ◯ Some children
- ◯ None of the children

Were people able to access Sub-health centre services?

- ◯ Not existant
- ◯ Accessible
- ◯ Accessible with good quality treatment

Were people able to access Primary Health Centre services

- ◯ Not close by
- ◯ Accessible
- ◯ Accessible with good quality treatment

Were people able to access Community Health Centre services?

- ◯ Difficult to access
- ◯ People are able to go
- ◯ People are able to go and has good quality treatment

Were people able to access District Hospital services?

- ◯ Difficult to access
- ◯ People are able to go
- ◯ People are able to go and has good quality treatment
