# Supplementary material for: Community-Based Approaches to Increase COVID-19 Vaccine Uptake and Demand: Lessons Learned from Four UNICEF-Supported Interventions
Source: Vaccines (Basel). 2023 Jun 30;11(7):1180. doi: 10.3390/vaccines11071180 (PMC10384848; doi:10.3390/vaccines11071180)
Supplement: Supplementary file 1 [file vaccines-11-01180-s001.zip › S2. Final_Monthly Data Tracker _ KoboToolbox.pdf]

Final_Monthly Data Tracker

Please collate and add up the data for the 8 hamlets together in this tracker

1.State

- ◯ Andhra Pradesh
- ◯ Bihar
- ◯ Chhattisgarh
- ◯ Gujarat
- ◯ Madhya Pradesh
- ◯ Odisha
- ◯ Rajasthan
- ◯ Tamil Nadu
- ◯ Telangana
- ◯ Uttar Pradesh
- ◯ West Bengal

2.District

3.Month

- ◯ November-December 2021

4.Number of district level stakeholder meetings done

____

4a.Designation of relevant officials/ stakeholders met during these district level meetings

____

5.Number of Block level stakeholder meetings done

____

5a.Designation of relevant officials/ stakeholders met during the block level meetings

____

6.Number of Panchayat level stakeholder meetings done (for integration with Gram Panchayat Plan)

____

6a.Designation of relevant officials/ stakeholders met during the panchayat level meetings

____

7.Number of hamlet level community meetings done (based on photographs with key points)

____

7a.Total participants in all hamlet level meetings: Cumulative

____

7b. Some key issues raised in hamlet level meetings (you can list them out together)

8.Number of letters written to stakeholders (based on copies of letters)

8a.Names of stakeholders to whom letters were written

9. Number of meetings held with village level frontline workers

10a. Number of Male adults (18+) identified who are willing to get vaccinated (based on your internal list)

10b. Number of Female adults (18+) identified who are willing to get vaccinated (based on your internal list)

10c. Number of Transgender/Non Binary adults (18+) identified who are willing to get vaccinated (based on your internal list)

11a.Number of Male adults (18+ years) supported with vaccination (based on your internal list)

11b.Number of Female adults (18+ years) supported with vaccination (based on your internal list)

11c.Number of Transgender/Non Binary adults (18+ years) supported with vaccination (based on your internal list)

12a. Number of boys (15-17 years) supported with vaccination (based on your internal list)

12b. Number of girls (15-17 years) supported with vaccination (based on your internal list)

12c. Number of transgender/non binary children (15-17 years) supported with vaccination (based on your internal list)

13. Number of vaccination camps organised or supported (based on photographs)

14.Please share if any innovative campaign or awareness building strategy was followed by you in any hamlet
