# Supplementary material for: Community-Based Approaches to Increase COVID-19 Vaccine Uptake and Demand: Lessons Learned from Four UNICEF-Supported Interventions
Source: Vaccines (Basel). 2023 Jun 30;11(7):1180. doi: 10.3390/vaccines11071180 (PMC10384848; doi:10.3390/vaccines11071180)
Supplement: Supplementary file 1 [file vaccines-11-01180-s001.zip › S3. Baseline_UNICEF_Vaccination survey Tool.pdf]

# COLLECT : Leave No One Behind Survey (CAB and Vaccination)

## A. Basic Details

**1. State**

______________________________

**2. District**

______________________________

**3. Block**

______________________________

**4. Panchayat**

______________________________

**5. Village**

______________________________

**6. Tola/Hamlet**

______________________________

**7. How many children between 12-17 years are there in the house?**

______________________________

**8. Community**

*Please carry out this survey only with SC/ST/Minority and NT/DNT households*

- ◯ SC
- ◯ ST
- ◯ OBC
- ◯ General

**9. Is the household from a DNT community?**

- ◯ Yes
- ◯ No

**10. Is the household from a Minority community**

- ◯ Yes
- ◯ No

**11. Name of the respondent**

______________________________

**12. Phone number to contact the household**

______________________________

## Vaccination Data

### » B. How many adult (18+) women in the family are:

**13.1 Not Vaccinated (Women 18+)**

______________________________

**13.2 Partially vaccinated / Only single dose (Women 18+)**

______________________________

**13.3 Fully vaccinated / Two doses (Women 18+)**

______________________________

### » C. How many adult (18+) men in the family are:

**14.1 Not Vaccinated (Men 18+)**

______________________________

**14.2 Partially vaccinated / Only single dose (Men 18+)**

______________________________

**14.3 Fully vaccinated / Two doses (Men 18+)**

______________________________

**15. Do you know where you can get the COVID-19 vaccine?**

- ○ Yes
- ○ No

## D. Reasons for non-vaccination

**16. If not, Then ask why vaccine is not taken?**

- [ ] Vaccine is not available/Shortage of vaccine
- [ ] 2nd dose is not due yet
- [ ] Price is high/Can't afford
- [ ] Facility is far-off/no one to take me
- [ ] Nearest facility is a private one
- [ ] Don't know where (facility) to go
- [ ] Long waiting time at facilities
- [ ] Don't have photo-ID document
- [ ] Fear of side-effects
- [ ] Not sure about Vaccines' efficacy
- [ ] Will change DNA
- [ ] May affect fertility of women
- [ ] Will be affective for a few months only
- [ ] Women stay at home so don't need on priority
- [ ] Old/senior citizens don't want to get vaccinated
- [ ] Female members are hesitant to get vaccinated
- [ ] Youth above 18 yrs. feel they have strong immunity
- [ ] Those who got vaccinated are also getting infected
- [ ] No/not many cases of COVID-19 infection in our locality/area/village
- [ ] Got COVID-19 positive after 1st dose so feel it is ineffective
- [ ] Got COVID-19 positive, so doctor/health worker advised not needed
- [ ] Other

**16.1 Please describe/mention other reasons**

---

## E. Focused Questions on PWDs

**17. Does the family have any persons with disability?**

- ○ Yes
- ○ No

**17.1 Have they been vaccinated?**

- ◯ Yes, First shot
- ◯ Yes, both shots
- ◯ Not vaccinated

**17.2 Reasons for No**

- ☐ 2nd dose is not due yet
- ☐ Facility is not accessible/no one to take me
- ☐ Fear of side-effects
- ☐ Long wait time at facilities
- ☐ Don't know where to go for vaccination
- ☐ Got COVID-19 positive, so doctor/health worker advised not needed
- ☐ Got COVID-19 positive after 1st dose so feel it is ineffective
- ☐ Vaccine is not available/Shortage of vaccine
- ☐ Price is high/Can't afford
- ☐ Nearest facility is a private one
- ☐ Not sure about Vaccines' efficacy
- ☐ Will change DNA
- ☐ May affect fertility of women
- ☐ Will be effective for a few months only
- ☐ They stay at home so don't need on priority
- ☐ Other

**17.3 Please describe/mention other reasons**

______________________________________________

## F. Focused questions on Pregnant Women

**18. Does the family have any pregnant women?**

- ◯ Yes
- ◯ No

**18.1 Have they been vaccinated?**

- ◯ Yes, First shot
- ◯ Yes, both shots
- ◯ Not vaccinated

**18.2 Reasons for No**

- [ ] 2nd dose is not due yet
- [ ] Facility is not accessible/no one to take me
- [ ] Fear of side-effects
- [ ] Long wait time at facilities
- [ ] Don't know where to go for vaccination
- [ ] Got COVID-19 positive, so doctor/health worker advised not needed
- [ ] Got COVID-19 positive after 1st dose so feel it is ineffective
- [ ] Vaccine is not available/Shortage of vaccine
- [ ] Price is high/Can't afford
- [ ] Nearest facility is a private one
- [ ] Not sure about Vaccines' efficacy
- [ ] Will change DNA
- [ ] May affect fertility of women
- [ ] Will be effective for a few months only
- [ ] Women stay at home so don't need on priority
- [ ] Other

**18.3 Please describe/mention other reasons**

---

## G. Focused questions on Transgender/ Non-Binary Identities

**19. Does the family have any Transgender/Non-binary persons?**

- ○ Yes
- ○ No

**19.1 Have they been vaccinated?**

- ○ Yes, First shot
- ○ Yes, both shots
- ○ Not vaccinated

**19.2 Reasons for No**

- [ ] 2nd dose is not due yet
- [ ] Facility is not accessible/no one to take me
- [ ] Fear of side-effects
- [ ] Was discriminated against at vaccination centre
- [ ] Long wait time at facilities
- [ ] Don't know where to go for vaccination
- [ ] Got COVID-19 positive, so doctor/health worker advised not needed
- [ ] Got COVID-19 positive after 1st dose so feel it is ineffective
- [ ] Vaccine is not available/Shortage of vaccine
- [ ] Price is high/Can't afford
- [ ] Nearest facility is a private one
- [ ] Not sure about Vaccines' efficacy
- [ ] Will change DNA
- [ ] May affect fertility
- [ ] Will be effective for a few months only
- [ ] Stay at home so don't need on priority
- [ ] Other

**19.3 Please describe/mention other reasons**

___
