# Supplementary material for: Community-Based Approaches to Increase COVID-19 Vaccine Uptake and Demand: Lessons Learned from Four UNICEF-Supported Interventions
Source: Vaccines (Basel). 2023 Jun 30;11(7):1180. doi: 10.3390/vaccines11071180 (PMC10384848; doi:10.3390/vaccines11071180)
Supplement: Supplementary file 1 [file vaccines-11-01180-s001.zip › S4. Endline Vaccination Survey 2 _ KoboToolbox.pdf]

Chhattisgarh COLLECT : Endline Vaccination Survey 2

A. Basic Details

1. State

- ◯ Chhattisgarh

2. District

- ◯ Bilaspur
- ◯ Gariaband
- ◯ Janjhgir Champa
- ◯ Korba
- ◯ Mungeli
- ◯ Rajnandgaon
- ◯ Balrampur

3. Block

4. Panchayat

5. Village

6. Tola/Hamlet

7. Community

Please carry out this survey only with SC/ST/Minority and NT/DNT households

- ◯ SC
- ◯ ST
- ◯ OBC
- ◯ General

8. Is the household from a DNT community?

- ◯ Yes
- ◯ No

9. Is the household from a Minority (Muslim) community? (Please ask the household members to reveal this information only if they are comfortable)

- ◯ Yes
- ◯ No
- ◯ No, from other minority community

9.1 In case from other minority community, please provide the name? (Please ask the household members to reveal this information only if they are comfortable)

____

10. Name of the respondent

____

11. Phone number to contact the household

____

12. Are there any children between the age of 12-14 years in this household?

- ◯ Yes
- ◯ No

13. Are there any children between the age of 15-17 years in this household?

- ◯ Yes
- ◯ No

Vaccination Data

» B. How many adult (18+) women in the family are:

13.1 Not Vaccinated (Women 18+)

____

13.2 Partially vaccinated / Only single dose (Women 18+)

____

13.3 Fully vaccinated / Two doses (Women 18+)

____

» C. How many adult (18+) men in the family are:

14.1 Not Vaccinated (Men 18+)

____

14.2 Partially vaccinated / Only single dose (Men 18+)

____

14.3 Fully vaccinated / Two doses (Men 18+)

____

15. Do you know where you can get the COVID-19 vaccine?

- ( ) Yes
- ( ) No

D. Reasons for non-vaccination

16. If not, Then ask why vaccine is not taken?

- [ ] Vaccine is not available/Shortage of vaccine
- [ ] 2nd dose is not due yet
- [ ] Price is high/Can't afford
- [ ] Facility is far-off/no one to take me
- [ ] Nearest facility is a private one
- [ ] Don't know where (facility) to go
- [ ] Long waiting time at facilities
- [ ] Don't have photo-ID document
- [ ] Fear of side-effects
- [ ] Not sure about Vaccines' efficacy
- [ ] Will change DNA
- [ ] May affect fertility of women
- [ ] Will be affective for a few months only
- [ ] Women stay at home so don't need on priority
- [ ] Old/senior citizens don't want to get vaccinated
- [ ] Female members are hesitant to get vaccinated
- [ ] Youth above 18 yrs. feel they have strong immunity
- [ ] Those who got vaccinated are also getting infected
- [ ] No/not many cases of COVID-19 infection in our locality/area/village
- [ ] Got COVID-19 positive after 1st dose so feel it is ineffective
- [ ] Got COVID-19 positive, so doctor/health worker advised not needed
- [ ] Other

16.1 Please describe/mention other reasons

____

E. Vaccination status of girls (15-17 years)

Number of girls between 15-17 years who have not been vaccinated at all

____

Number of girls between 15-17 years who have been partially vaccinated (received one COVID vaccine dose)

____

Number of girls between 15-17 years who have been fully vaccinated (received both COVID vaccine dose)

____

F. Vaccination status of boys (15-17 years)

Number of boys between 15-17 years who have not been vaccinated at all

____

Number of boys between 15-17 years who have been partially vaccinated (received one COVID vaccine dose)

____

Number of boys between 15-17 years who have been fully vaccinated (received both COVID vaccine dose)

____

If not, Then ask why vaccine is not taken by children 15-17 years old?

- [ ] 2nd dose not due yet
- [ ] Fear of side effects
- [ ] Parents do not want to sign consent letter
- [ ] Vaccine could affect female fertility
- [ ] Don't have photo-ID document
- [ ] Facility is far-off/no one to take me
- [ ] Price is high/Can't afford
- [ ] Feel children are young so have strong immunity
- [ ] girl child stays at home so does not need vaccine
- [ ] No/not many cases of COVID-19 infection in our locality/area/village
- [ ] Got COVID-19 positive after 1st dose so feel it is ineffective
- [ ] Got COVID-19 positive, so doctor/health worker advised not needed
- [ ] Had difficulty in registering on COWIN app
- [ ] Vaccine shortage/unavailable
- [ ] Parents out for work/do not have time to take them to the centre
- [ ] Others

Please describe/mention other reasons

____

G. Vaccination status of girls (12-14 years)

Number of girls between 12-14 years who have not been vaccinated at all

____

Number of girls between 12-14 years who have been partially vaccinated (received one COVID vaccine dose)

____

Number of girls between 12-14 years who have been fully vaccinated (received both COVID vaccine dose)

____

H. Vaccination status of boys (12-14 years)

Number of boys between 12-14 years who have not been vaccinated at all

____

Number of boys between 12-14 years who have been partially vaccinated (received one COVID vaccine dose)

Number of boys between 12-14 years who have been fully vaccinated (received both COVID vaccine dose)

If not, Then ask why vaccine is not taken by children 12-14 years old?

- ☐ 2nd dose not due yet
- ☐ Fear of side effects
- ☐ Parents do not want to sign consent letter
- ☐ Vaccine could affect female fertility
- ☐ Don't have photo-ID document
- ☐ Facility is far-off/no one to take me
- ☐ Price is high/Can't afford
- ☐ Feel children are young so have strong immunity
- ☐ girl child stays at home so does not need vaccine
- ☐ No/not many cases of COVID-19 infection in our locality/area/village
- ☐ Got COVID-19 positive after 1st dose so feel it is ineffective
- ☐ Got COVID-19 positive, so doctor/health worker advised not needed
- ☐ Had difficulty in registering on COWIN app
- ☐ Vaccine shortage/unavailable
- ☐ Parents out for work/do not have time to take them to the centre
- ☐ Others

Please describe/mention other reasons

E. Focused Questions on PWDs

17. Does the family have any persons with disability?

- ○ Yes
- ○ No

17.1 Have they been vaccinated?

- ○ Yes, First shot
- ○ Yes, both shots
- ○ Not vaccinated

17.2 Reasons for No

- ☐ 2nd dose is not due yet
- ☐ Facility is not accessible/no one to take me
- ☐ Fear of side-effects
- ☐ Long wait time at facilities
- ☐ Don't know where to go for vaccination
- ☐ Got COVID-19 positive, so doctor/health worker advised not needed
- ☐ Got COVID-19 positive after 1st dose so feel it is ineffective
- ☐ Vaccine is not available/Shortage of vaccine
- ☐ Price is high/Can't afford
- ☐ Nearest facility is a private one
- ☐ Not sure about Vaccines' efficacy
- ☐ Will change DNA
- ☐ May affect fertility of women
- ☐ Will be effective for a few months only
- ☐ They stay at home so don't need on priority
- ☐ Other

17.3 Please describe/mention other reasons

F. Focused questions on Pregnant Women

18. Does the family have any pregnant women?

- ○ Yes
- ○ No

18.1 Have they been vaccinated?

- ○ Yes, First shot
- ○ Yes, both shots
- ○ Not vaccinated

18.2 Reasons for No

- ☐ 2nd dose is not due yet
- ☐ Facility is not accessible/no one to take me
- ☐ Fear of side-effects
- ☐ Long wait time at facilities
- ☐ Don't know where to go for vaccination
- ☐ Got COVID-19 positive, so doctor/health worker advised not needed
- ☐ Got COVID-19 positive after 1st dose so feel it is ineffective
- ☐ Vaccine is not available/Shortage of vaccine
- ☐ Price is high/Can't afford
- ☐ Nearest facility is a private one
- ☐ Not sure about Vaccines' efficacy
- ☐ Will change DNA
- ☐ May affect fertility of women
- ☐ Will be effective for a few months only
- ☐ Women stay at home so don't need on priority
- ☐ Other

18.3 Please describe/mention other reasons

G. Focused questions on Transgender/ Non-Binary Identities

19. Does the family have any Transgender/Non-binary persons?

- ○ Yes
- ○ No

19.1 Have they been vaccinated?

- ○ Yes, First shot
- ○ Yes, both shots
- ○ Not vaccinated

19.2 Reasons for No

- [ ] 2nd dose is not due yet
- [ ] Facility is not accessible/no one to take me
- [ ] Fear of side-effects
- [ ] Was discriminated against at vaccination centre
- [ ] Long wait time at facilities
- [ ] Don't know where to go for vaccination
- [ ] Got COVID-19 positive, so doctor/health worker advised not needed
- [ ] Got COVID-19 positive after 1st dose so feel it is ineffective
- [ ] Vaccine is not available/Shortage of vaccine
- [ ] Price is high/Can't afford
- [ ] Nearest facility is a private one
- [ ] Not sure about Vaccines' efficacy
- [ ] Will change DNA
- [ ] May affect fertility
- [ ] Will be effective for a few months only
- [ ] Stay at home so don't need on priority
- [ ] Other

19.3 Please describe/mention other reasons

H. Decision Making and Sources of Informaion

20.1 Who in the family decides about who all should get vaccinated?

- [ ] Individual decision of each member
- [ ] Head of the household-male
- [ ] Head of the household-female
- [ ] Male member(s)
- [ ] Female member(s)
- [ ] Head/influencer of the community

20.2 How are you accessing information on vaccination?

- [ ] TV News
- [ ] Social Media (Whatsapp/Facebook etc.)
- [ ] Print Media (Newspaper/Magazine)
- [ ] Government Officials
- [ ] Health workers & health facilities
- [ ] Others

20.3 In case selected others, please provide names of the other sources

____
